# Reprogrammed CD4^+^ T Cells That Express FoxP3^+^ Control Inhibitory Antibody Formation in Hemophilia A Mice

**DOI:** 10.3389/fimmu.2019.00274

**Published:** 2019-02-20

**Authors:** Roland W. Herzog, Veronica Kuteyeva, Rania Saboungi, Cox Terhorst, Moanaro Biswas

**Affiliations:** ^1^Herman B Wells Center for Pediatric Research, Indiana University School of Medicine, Indianapolis, IN, United States; ^2^Department of Pediatrics, College of Medicine, University of Florida, Gainesville, FL, United States; ^3^Division of Immunology, Beth Israel Deaconess Medical Center (BIDMC), Harvard Medical School, Boston, MA, United States

**Keywords:** hemophilia A, tolerance, FoxP3, treg, cell therapy, immunotherapy

## Abstract

Coagulation Factor VIII (FVIII) replacement therapy in hemophilia A patients is complicated by the development of inhibitory antibodies, which often render the treatment ineffective. Previous studies demonstrated a strong correlation between induction of regulatory T cells (Treg) and tolerance to the therapeutic protein. We, therefore, set out to evaluate whether the adoptive transfer of FVIII-specific CD4^+^ Treg cells prevents inhibitor response to FVIII protein therapy. To this end, we first retrovirally transduced FoxP3^+^ into FVIII-specific CD4^+^ cells, which resulted in cells that stably express FoxP3, are phenotypically similar to peripherally induced Tregs and are antigen specific suppressors, as judged by *in vitro* assays. Upon transfer of the FVIII-specific CD4^+^ FoxP3^+^ cells into hemophilia A mice, development of inhibitory antibodies in response to administering FVIII protein was completely suppressed. Suppression was extended for 2 months, even after transferred cells were no longer detectable in the secondary lymphoid organs of recipient animals. Upon co-transfer of FoxP3^+^-transduced cells with the B cell depleting anti-CD20 into mice with pre-existing inhibitory antibodies to FVIII, the escalation of inhibitory antibody titers in response to subsequent FVIII protein therapy was dramatically reduced. We conclude that reprogramed FoxP3 expressing cells are capable of inducing the *in vivo* conversion of endogenous FVIII peripheral Tregs, which results in sustained suppression of FVIII inhibitors caused by replacement therapy in recipient hemophilia A animals.

## Introduction

Hemophilia A is one of the most common X-linked recessive disorders, affecting 1 in 5,000 male births worldwide. This blood clotting disorder is caused by mutations in the factor VIII (FVIII) gene, leading to a deficiency in FVIII production. Depending on the causative mutation, FVIII production can be either completely absent or may vary up to 5–40% of normal factor activity in blood, thus manifesting as severe, moderate, or mild forms of the disorder (1). Replacement therapy with plasma derived or recombinant FVIII infusions are the standard of care for managing hemophilia A patients, and exciting strides have been made in developing gene therapy for hemophilia A and B that have the potential to provide a lifelong cure (2–5).

The most challenging issue with conventional factor replacement therapy in the treatment of hemophilia A is the development of antibodies against infused FVIII, which occur in 25–30% of severe hemophilia A patients. Inhibitor prevalence is influenced by severity of the disorder and is often associated with large deletions/inversions in the *F8* gene, which results in the lack of FVIII formation (6). Inhibitors render factor replacement therapy ineffective and can present a high risk of morbidity and mortality (7). Immune tolerance induction (ITI) for the eradication of inhibitors via frequent and high dose exposure to FVIII concentrates for a prolonged period is expensive and not always successful, especially in severe hemophilic patients (8). Mechanisms for tolerance induction by ITI are not clearly known but may include T effector cell (T_eff_) exhaustion/anergy, inhibition of FVIII-specific memory B-cell differentiation, or induction of regulatory T cells (Tregs) (9, 10). Conversely, there is also very little information on the immune interactions that lead to the development of inhibitors, although it has been described to be a T helper dependent process involving antigen uptake and presentation that requires the co-operation of multiple macrophage, dendritic cell or B cell subsets of antigen presenting cells (APC) (11–15).

Multiple studies have demonstrated that tolerance to replacement FVIII protein is strongly modulated by Tregs (16, 17). Co-administration of FVIII with drugs such as sirolimus (rapamycin), alone or in combination with cytokines such as IL-10 or Flt3L have been shown to induce and/or expand CD4^+^CD25^+^FoxP3^+^ Tregs, either through specific deletion of CD4^+^ T_eff_ cells which are more sensitive to mTOR inhibition, or selective expansion of plasmacytoid dendritic cells (pDCs) (18–20). Similar results have been obtained by treatment with IL-2/anti-IL-2 complexes or oral anti-CD3 treatment (21–24).

Tregs can be naturally occurring (central or thymic), with specificity mainly toward endogenous “self” antigens, or peripherally derived (extra-thymically induced), with specificity to exogenously introduced antigens (25). The lack of endogenous FVIII protein expression in severe hemophilia A patients with large mutations in the *F8* gene results in ineffective FVIII Treg induction and T_eff_ escape during thymic selection, reflected in the higher rate of inhibitor development for these patients. Therefore, there is great interest in re-establishing tolerance to FVIII in these cases.

Cellular therapy with Tregs, either freshly isolated or *ex vivo* expanded, is a promising approach for tolerance induction, as has been demonstrated in several clinical trials for autoimmune disorders and in transplant studies (26–29). While autologous Tregs of a polyclonal specificity are effective, as observed in a study in hemophilia A mice (30), it is anticipated that antigen-specific Tregs would be more effective at much lower frequencies, with a significantly reduced risk for off-target suppression (31). In this study, we hypothesized that forced FoxP3 expression in conventional/effector CD4^+^ T cells (T_conv_/T_eff_) from hemophilia A mice that were immunized with FVIII would yield an enriched pool of FVIII specific suppressor Treg-like cells. We examined the phenotype of these cells, and stability of FoxP3 expression over time, and were able to suggest a potential role for lasting suppression by a mechanism of conversion of T_eff_ cells into antigen-specific endogenous Tregs. Adoptively transferred FoxP3 expressing cells from FVIII immunized mice (FoxP3^FVIII^) were able to successfully prevent inhibitor formation in previously untreated hemophilia A mice and, when applied as combination therapy with a B-cell depleting antibody (anti-mCD20), were able to reverse established inhibitors to FVIII. This study therefore underlines the potential of gene-engineered cells with Treg function to provide specific and lasting suppression. This cell-based tolerance approach can potentially act as stand-alone therapy or can complement conventional ITI to re-establish tolerance to FVIII replacement therapy.

## Methods

### Mice

All wt animals used in the experiments were 8–10-week-old male mice of the BALB/c [H-2^d^] background, which were purchased from Jackson Laboratories (Bar Harbor, ME). DO11.10-tg Rag2^−/−^ mice with a transgenic T cell receptor specific for the amino acid sequence 323–339 of chicken ovalbumin (OVA), presented by MHCII I-A^d^, were originally obtained from Taconic (Hudson, NY). Hemophilia A mice with a deletion in exon 16 of the *F8* gene (BALB/c *F8*e16^−/−^) were originally provided by Dr. David Lillicrap (Queens, Ontario, Canada).

### Plasmids and Transduction

MIGR-mFoxP3 was a gift from Dan Littman (Addgene plasmid # 24067). MIGR1 (IRES-GFP) was a gift from Warren Pear (Addgene plasmid # 27490). Both plasmids are based on the murine stem cell virus (MSCV) expression system. Transfer plasmids were introduced into the PlatE ecotropic retroviral packaging cell line (Cell Biolabs Inc, San Diego, CA) using the Viafect transfection reagent (Promega, Madison, WI) and supernatants were collected after 48 h. CD4^+^CD25^−^ T_conv_ cells were magnetically isolated (Miltenyi Biotec) and pre-activated for 48 h with a 2:1 ratio of CD3/28 mouse activator beads (Dynabeads, Invitrogen) to cells. Cells were cultured in RPMI-1640 media (Life Technologies) supplemented with 10% fetal bovine serum (Atlanta Biologicals, Norcross, GA), 10,000 IU/ml penicillin, 10 mg/ml streptomycin, 1X GlutaMAX-1, 1 mmol/l sodium pyruvate, 10 mmol/l HEPES, 1X non-essential amino acids and 10 μmol/l 2-mercaptoethanol. Recombinant hIL-2 (Proleukin/aldesleukin, Prometheus Therapeutics and Diagnostics, San Diego, CA) was added at a concentration of 200 IU/ml. Activated T_eff_ cells were retrovirally transduced by spinoculation at 1,200 g for 90 min into retronectin (Takara) coated plates. GFP^+^ cells, representing FoxP3 (MIGR-mFoxP3 IRES-GFP) or GFP only (MIGR IRES-GFP) transduced populations of CD4^+^ T cells, were purified using the FACSAria II cell sorter (BD Biosciences).

### Reagents

Purified CD16/32 (Fc Block), CD3 (PerCP-Cy5.5), GITR (V500) antibodies were from BD Biosciences (San Jose, CA); CD39 (eFluor450), CTLA-4 (PE), FoxP3 (eFluor660) antibodies were purchased from eBioscience (San Diego, CA); CD25 (BV605), TNFR2 (PE), CD127 (PerCP-Cy5.5), CD62L (APC/Cy7), anti-GFP (A488), CD4 (BV421), CD4 (A700) antibodies were from Biolegend (San Diego, CA). Mouse neuropilin-1 antibody (PE) was from R&D Systems (Minneapolis, MN). Mouse CD4^+^ T cell isolation kit, CD4^+^CD25^+^ regulatory T cell isolation kits were from Miltenyi Biotec (Auburn, CA). OVA peptide (323–339) was synthesized by Anaspec (Fremont, CA). Recombinant human B domain deleted (BDD) FVIII (Xyntha) was from Pfizer (New York, NY). FVIII deficient plasma was from Haematologic Technologies (Essex Junction, VT). Anti-mCD20 IgG2a subtype (clone 2B8) was a kind gift from Biogen (Cambridge, MA).

### Analysis of Plasma Samples

Plasma samples were collected by retro-orbital eye bleed into 0.38% sodium citrate buffer and analyzed using a modified activated partial thromboplastin time assay (aPTT). Inhibitory antibodies to FVIII were measured by Bethesda assay as described (32). One Bethesda unit (BU) is defined as the reciprocal of the dilution of test plasma at which 50% of FVIII or FIX activity is inhibited. Measurements were carried out in a Diagnostica Stago STart Hemostasis Analyzer (Parsippany, NJ). Enzyme-linked immunosorbent assay (ELISA)-based measurements of IgG1 antibodies to FVIII were carried out as described (32).

### Adoptive Transfer Studies, Inhibitor Establishment, or Reversal

To generate a source of CD4^+^ T cells that were enriched for specificity toward FVIII, BALB/c F8e16^−/−^ hemophilia A (HA) mice were subcutaneously immunized with 1IU FVIII/mouse delivered in adjuvant (Sigma Adjuvant System oil, Sigma-Aldrich, St. Louis, MO). A booster immunization was administered 2 weeks later. Immunized mice developed high titer antibodies (~40 BU/ml) to FVIII. 2 × 10^6^ FoxP3 transduced cells from either FVIII immunized mice (FoxP3^FVIII^) or naïve animals (FoxP3^naive^) were adoptively transferred into recipient HA mice (n = 5–7). For prevention studies, recipient BALB/c F8e16^−/−^ HA mice received either FoxP3^FVIII^, FoxP3^naive^, GFP^FVIII^, or nothing. Mice then received 1.5 IU of BDD-FVIII by weekly tail-vein injections for 2 months. Plasma samples were analyzed at 1 and 2 months for inhibitor development. For reversal studies, inhibitors were established by 1.5 IU BDD-FVIII injections for 1 month. Mice then received either FoxP3^FVIII^ cells, mCD20 IgG2a antibody (IV, 1, and 3 weeks after inhibitor establishment) or a combination of FoxP3^FVIII^ cells and mCD20 IgG2a antibody, following which, weekly BDD-FVIII injections were continued. Plasma samples were analyzed at 1, 2, 3- and 4-months post-inhibitor establishment.

### Phenotypic Characterization of FoxP3 Transduced Cells

FoxP3 or GFP transduced cells were evaluated for expression of Treg associated markers. Since retroviral transduction requires pre-activation of CD4^+^ T cells, which can upregulate several phenotypic markers in a non-specific manner, we attempted to analyze cells under physiological conditions. For this, 2 × 10^6^ FoxP3 or GFP transduced cells were adoptively transferred into wt BALB/c mice. Spleens were harvested after 48 h and GFP^+^ cells were identified and phenotyped. Endogenous FoxP3^+^ Tregs from recipient wt BALB/c mice were used as a standard to compare the difference in expression of various markers. For intracellular staining, cells were pre-fixed in 2% paraformaldehyde to retain GFP expression and intracellular staining was performed using the FoxP3 staining kit (eBioscience, San Diego, CA). Briefly, ~1 × 10^6^ cells in a volume of 100 μl were blocked with CD16/32 for 15 min and surface labeled with antibodies at recommended concentrations. Fixation-permeabilization carried out as required and intracellular antibodies were added. Samples were acquired on the Fortessa flow cytometer (BD Biosciences) and analyzed using FCS express 6 (DeNovo Software, Los Angeles, CA).

### *In vitro* Suppression Assay

To assay for *in vitro* suppression of polyclonally activated cells, CD4^+^CD25^−^ responder cells from spleens of DO11.10-tg Rag2^−/−^ mice were isolated, labeled with 3–5 μmol/l CellTrace Violet (Invitrogen, Carlsbad, CA) and cultured with CD4^−^ total splenocytes. DO11.10-tg Rag2^−/−^ GFP^+^ FoxP3 transduced CD4^+^ T cells were added at various Treg: T responder ratios and cells were cultured for 72 h at 37°C. Dilution of the CellTrace Violet label in GFP^−^ proliferating responder cells (T_eff_) was quantified. Proliferation was determined by quantifying CellTrace Violet fluorescence intensity relative to a parent population of unstimulated responder cells (0% proliferation) and stimulated cells incubated without Treg (100% proliferation). Percentage of CD4^+^ responder T cell proliferation was also determined using ModFit LT analysis on FCS Express 6.

### *In vivo* Conversion Assay

FACS-purified DO11.10-tg Rag2^−/−^ GFP^+^ FoxP3 transduced CD4^+^ T cells were labeled with 3–5 μmol/l CellTrace Violet (Invitrogen, Carlsbad, CA) and adoptively transferred into cohorts of recipient DO11.10-tg Rag2^−/−^ mice (n = 4) that naturally lack endogenous Tregs. Recipient mice subsequently received 50 μg OVA^323−339^ peptide, 3x/week for 2 weeks, delivered via i.p. injection. Control mice received no treatment. One group of mice received only FoxP3^OVA^ cells, while another received only OVA^323−339^ peptide and no cells. The 4th group received both FoxP3^OVA^ cells, as well as OVA^323−339^ peptide injections. *In vivo* conversion of endogenous OVA specific CD4^+^ T effectors into FoxP3^+^ Tregs was assessed at the end of the 2-week period. Induced endogenous Tregs were distinguished from adoptively transferred donor cells by the lack of GFP and CellTrace Violet label.

### Statistical Analysis

Statistical significance was determined using either student's *T*-test, 1-way or 2-way ANOVA with GraphPad Prism 7 software (La Jolla, CA). Values at *P* < 0.05 were deemed significant and indicated as follows: ^*^P < 0.05, ^**^P < 0.01, ^***^P < 0.001. For some samples, normality was assessed with the Shapiro-Wilk normality test. Non-parametric analyses were carried out using the Kruskal Wallis test. Difference in proportions of mice that developed inhibitors was determined using Fisher's exact test.

## Results

### Stably Transduced FoxP3^+^ CD4^+^ T Cells Acquire *in vitro* Suppressor Functions

**Figure 1:**
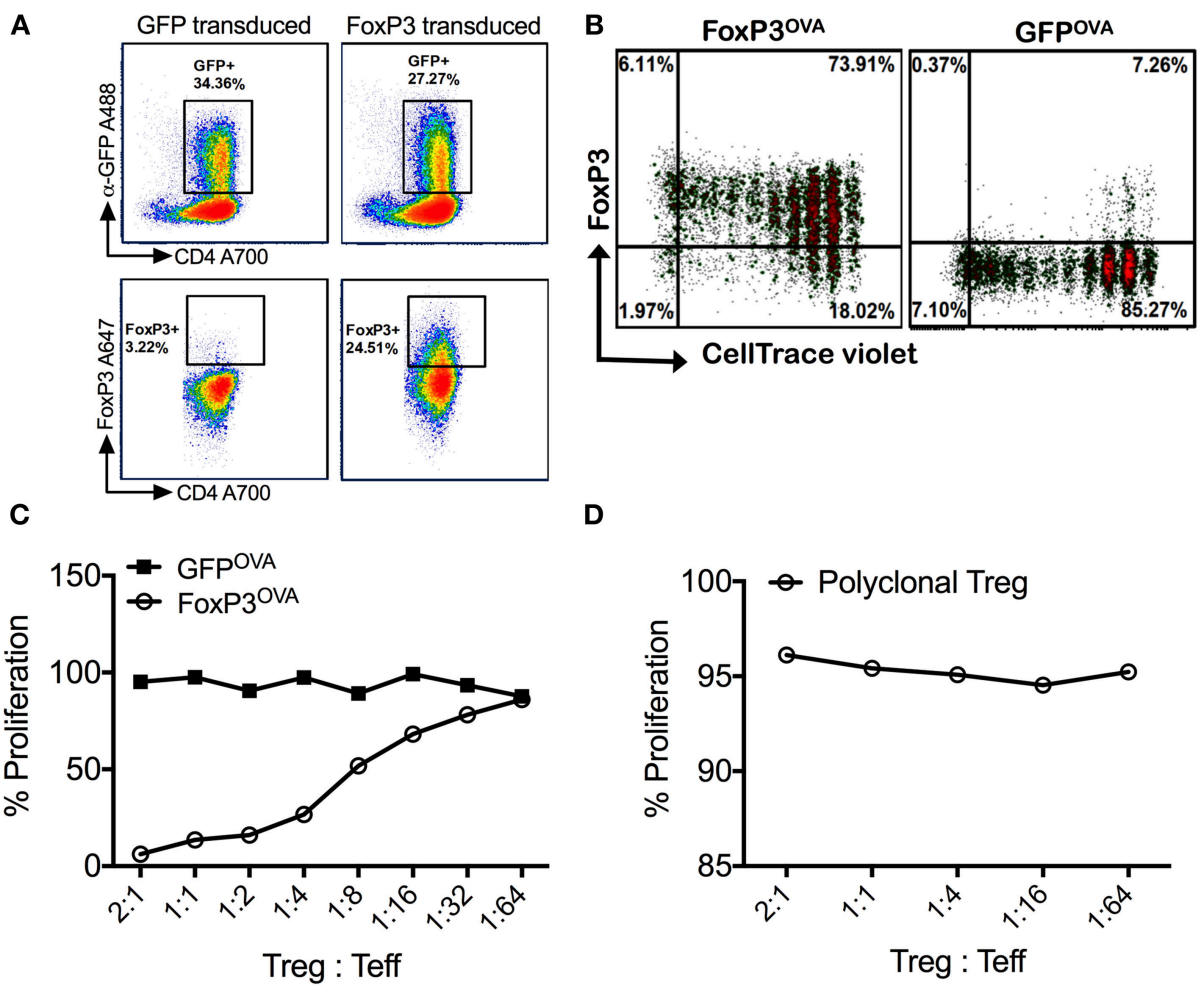
Retrovirally transduced cells stably express FoxP3 and are suppressive *in vitro*. **(A)** Isolated splenic murine CD4^+^CD25^−^ cells transduced with the MIGR-mFoxP3 vector strongly expressed GFP (upper panel), which correlated with intracellular FoxP3 expression (lower panel). Cells transduced with the control vector MIGR1 expressed only GFP. **(B)** FoxP3 transduced cells from DO11.10 Rag2^−/−^ mice were labeled with CellTrace Violet, adoptively transferred into wt BALB/c mice and recipients were injected with 100μg OVA^323−339^ peptide by the *i.p*. route. Spleens were recovered after 3 days. Robust proliferation of donor transgenic cells (FoxP3^OVA^) without compromise in FoxP3 expression was observed. Control DO11.10^+^ cells (GFP^OVA^) proliferated in response to OVA^323−339^ peptide administration but did not express FoxP3. **(C)** FoxP3^OVA^ cells strongly suppressed the proliferation of CellTrace Violet labeled DO11.10^+^ CD4^+^CD25^−^ responder cells cultured with CD4^−^ total splenocytes and stimulated with OVA^323−339^ peptide. Control GFP transduced cells (GFP^OVA^) or **(D)** freshly isolated tTregs of a polyclonal specificity did not suppress the proliferation of OVA^323−339^ stimulated responder T cells. Data is a single representative of at least 2 independent experiments.

Flow cytometric analysis of retrovirally transduced murine CD4^+^CD25^−^ T_conv_ cells revealed high expression of FoxP3, which directly correlated with GFP^+^ cells (Figure 1A). Transduction efficacies ranged from 25–60%. CD4^+^CD25^−^ Tconv cells transduced with the control vector only expressed GFP, without concurrent FoxP3 expression (Figure 1A). FoxP3 transduced CD4^+^ T cells were shown to undergo antigen specific proliferation without loss of FoxP3 expression *in vivo*. For this, we used DO11.10 Rag2^−/−^ mice, which lack mature B or T lymphocytes or endogenous Tregs but are transgenic for CD4^+^ T cells with OVA^323−339^ specificity. Donor FoxP3 transduced CD4^+^CD25^−^ cells from DO11.10 Rag2^−/−^ mice (FoxP3^OVA^), when adoptively transferred into recipient wt BALB/c mice and challenged with OVA^323−339^ peptide, underwent robust proliferation, as observed by dilution of the cell dye label, without compromise in FoxP3 expression (Figure 1B). This established that under non-inflammatory conditions, FoxP3 transduced cells can proliferate in an antigen dependent manner while stably retaining FoxP3 expression.

FoxP3 transduced DO11.10 CD4^+^ T cells were also shown to be functionally suppressive in an antigen specific manner *in vitro*. OVA^323−339^ peptide specific proliferation of responder DO11.10 CD4^+^CD25^−^ T cells was potently suppressed on addition of FoxP3 transduced cells of the same specificity (FoxP3^OVA^) (Figure 1C). Suppression was still strongly evident at a Treg:T_eff_ (responder) cell ratio of 1:16, indicating that FoxP3 transduction alone is capable of producing cells with Treg function. Control GFP transduced OVA specific cells (GFP^OVA^) did not suppress at any ratio (Figure 1C). To test if the observed suppression was antigen specific, the same assay was carried out with freshly isolated thymic Tregs (tTregs, Figure 1D). Because non-specific polyclonal tTregs failed to suppress the proliferation of OVA^323−339^ stimulated T_eff_ cells, we conclude that suppression by FoxP3 transduced cells in response to antigen is TCR specific, and not a result of a bystander effect.

### *In vivo* Treg Phenotype of Re-programmed FoxP3^+^ CD4^+^ T Cells

**Figure 2:**
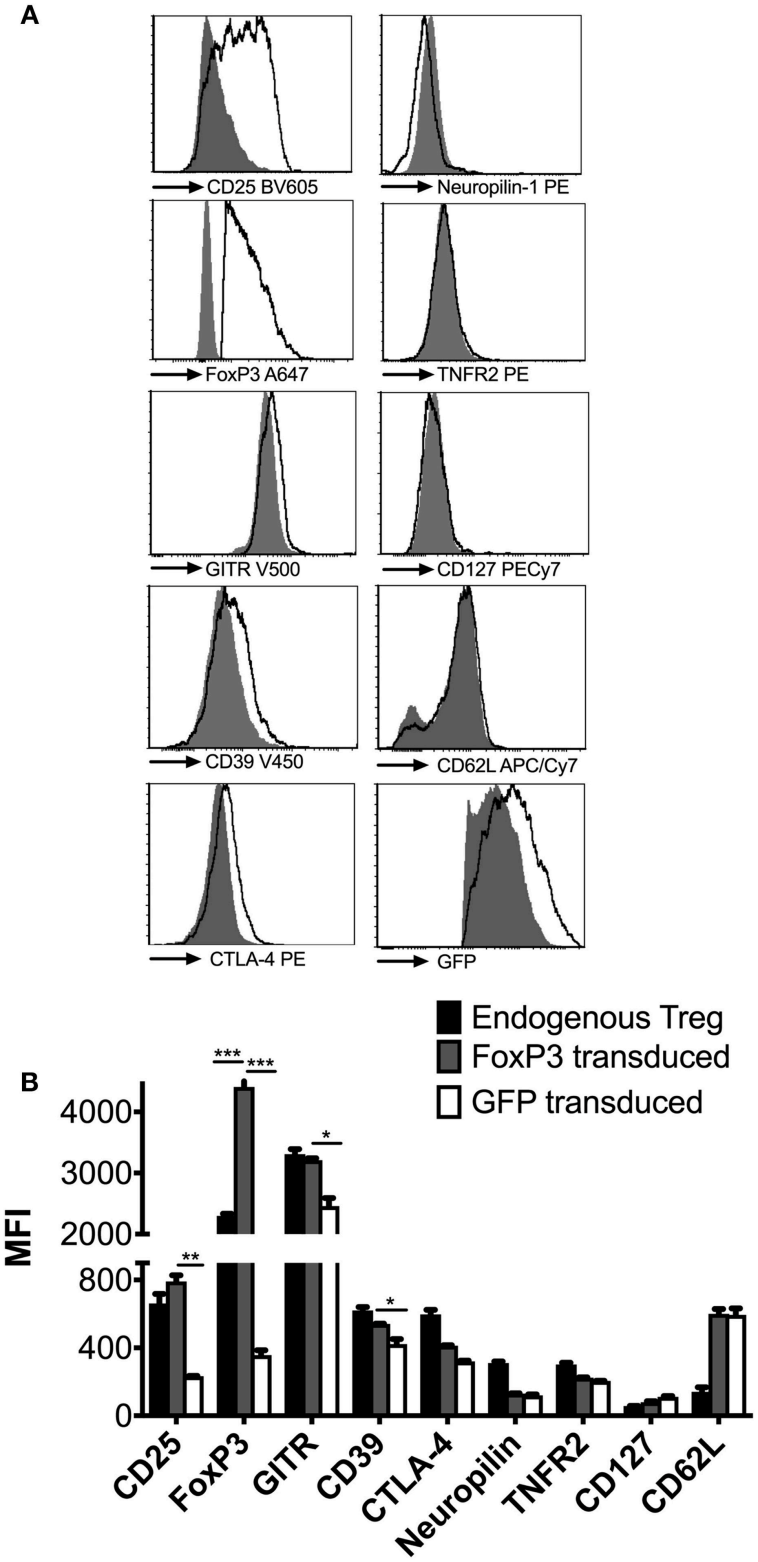
Phenotypic analysis of reprogrammed FoxP3 expressing Treg like cells. **(A)** FoxP3 transduced cells were adoptively transferred into wt BALB/c mice and re-isolated 2 days later to characterize them under *in vivo* conditions (empty histograms). Control GFP vector transduced cells (filled histograms) were also phenotyped for comparison. Expression levels of CD25, FoxP3, GITR, CD39, CTLA-4, NRP-1, TNFR2, CD127, CD62L, and GFP were quantified. **(B)** Median fluorescent intensity (MFI) of the above markers were compared between endogenous tTreg (black bars), FoxP3 transduced cells (gray bars) and GFP transduced cells (white bars). Data is representative of 2 samples and at least 2 independent experiments. ^*^P < 0.05, ^**^P < 0.01, ^***^P < 0.001.

To characterize the phenotype of the reprogrammed cells under physiological conditions, 2 × 10^6^ FoxP3 or GFP transduced cells were adoptively transferred into *wt* BALB/c mice and re-isolated from spleens after 48 h. FoxP3 transduced CD4^+^ T cells acquired a Treg-like phenotype, with high expression of CD25 and GITR, low expression of IL-7Ra chain (CD127), and FoxP3 expression levels that were higher than endogenous tTregs (Figures 2A,B). Expression of CD39 and CTLA-4 was higher than in CD4^+^ T cells transduced with GFP only, although expression levels did not reach those observed on endogenous tTregs. It has previously been observed that CTLA-4 expression is independent of FoxP3 (33, 34), which might explain the lack of correlation with FoxP3 overexpression. Furthermore, we had earlier shown that CTLA-4 is highly expressed in *ex vivo* expanded tTregs, which is a function of prolonged CD3/CD28 activation of these cells (30). FoxP3 transduced CD4^+^ T cells also lacked the Neuropilin-1 marker, which is generally associated with centrally derived tTregs in mice, (35, 36), thus confirming their induced nature (Figures 2A,B). Thus, reprogrammed FoxP3^+^ CD4^+^ T cells maintain a Treg phenotype *in vivo* and *in vitro*.

### FVIII-Specific Reprogrammed FoxP3^+^ Treg Cells Prevent Inhibitor Formation in Hemophilia A Mice

**Figure 3:**
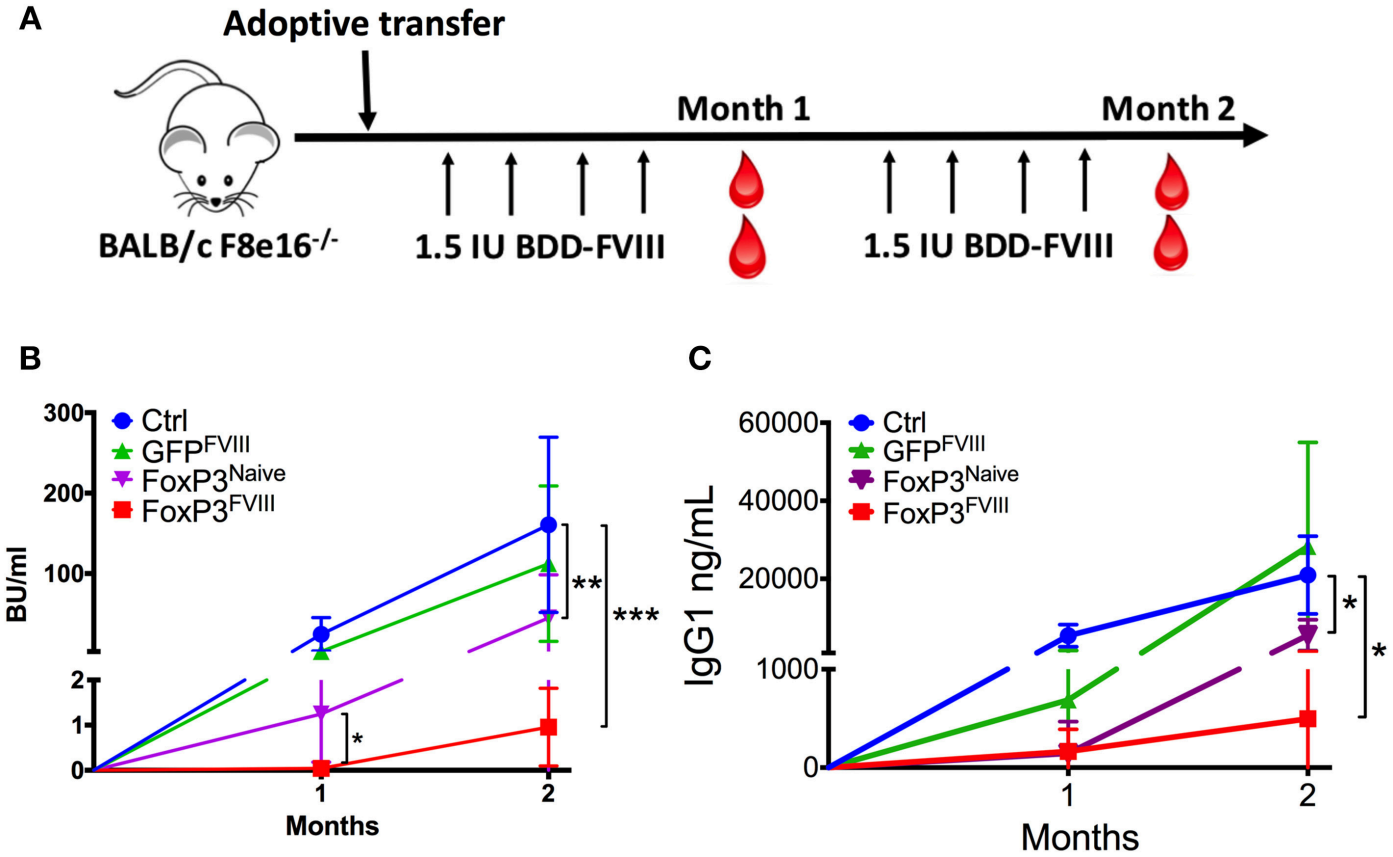
Prevention of inhibitor formation by FoxP3^FVIII^ cells. **(A)** Experimental timeline. BALB/c *F8*e16^−/−^ mice were transplanted with 2 × 10^6^ FoxP3 transduced cells from FVIII immunized mice (FoxP3^FVIII^). This was immediately followed by weekly intravenous injections with BDD-FVIII. Blood was collected at Months 1 and 2 for inhibitor assessment. **(B)** Inhibitor titers (BU/ml) were assessed at specific time points by the Bethesda assay. Experimental groups tested were control mice that received no treatment, FoxP3 transduced cells from naïve mice (FoxP3^Naï*ve*^), GFP transduced cells from FVIII immunized mice (GFP^FVIII^), and FoxP3 transduced cells from FVIII immunized mice (FoxP3^FVIII^). **(C)** Anti-FVIII IgG1 titers (ng/ml) at Months 1 and 2 were tested by ELISA. Data are average ±SD. Statistically significant differences are indicated for each time point. ^*^P < 0.05, ^**^P < 0.01, ^***^P < 0.001.

To determine whether engineered FoxP3^+^ CD4^+^ T cells could suppress the development of inhibitors to FVIII, BALB/c *F8*e16^−/−^ mice with no intrinsic FVIII expression were adoptively transferred with 2 × 10^6^ FoxP3 transduced cells. In order to simulate protein replacement therapy, mice were treated with 8 weekly *i.v*. injections with 1.5 IU BDD-FVIII protein (Figure 3A). Donor FoxP3^+^ cells were derived from mice immunized with BDD-FVIII in adjuvant, in order to enrich for FVIII specific T_eff_ cells that could be reprogrammed into engineered Tregs (FoxP3^FVIII^). FoxP3 transduction resulted in 50–60% GFP+ cells, which were further purified by cell sorting (Supplementary Figure 1). As shown in Figure 3B, FoxP3 transduced cells completely prevented the formation of functional inhibitory antibodies in response to repeated administrations of BDD-FVIII. Tolerance was sustained for 2 months of BDD-FVIII exposure (Month 1: **0.04** ± 0.02, Month 2: **0.95** ± 0.49 BU/ml). Control mice, which did not receive FoxP3 transduced cells, developed high inhibitor titers (Month 1: **24.45 ±** 7.8, Month 2: **160.59** ± 4.1 BU/ml). Similarly, CD4^+^ from FVIII immunized mice that were transduced with control GFP vector (GFP^FVIII^) were unable to mediate tolerance (Month 1: **3.0** ± 1.9, Month 2: **112.34** ± 43.1 BU/ml).

To confirm that enrichment of antigen specific cells in the FoxP3^FVIII^ population contributed favorably to suppression, one group of mice received FoxP3 transduced cells from naïve mice that had not been pre-exposed to FVIII. These FoxP3^Naive^ cells were suppressive at the 1-month time point (1.28±0.02 BU/ml), probably due to a non-specific regulatory effect at the time of BDD-FVIII introduction. However, this suppressive effect by FoxP3^Naive^ cells was significantly lower at the 1-month time point as compared to FoxP3^FVIII^ recipient mice (*p* = 0.018). The proportion of animals that developed inhibitors was also significantly greater at the 1-month time point in mice that received FoxP3^Naive^ cells (60% vs. 0%, *p* = 0.045). Furthermore, the observed tolerogenic effect conferred by the FoxP3^Naive^ cell population was not long-lasting, and high-titer inhibitors (>5BU/ml) developed in 80% of the mice by Month 2 (**44.8** ± 23 BU/ml), which was in contrast to the sustained tolerance exerted by FoxP3^FVIII^ cells (Month 2: **0.95** ± 0.49 BU/ml). Therefore, using FVIII experienced T cells for FoxP3 gene transfer resulted in more consistent and durable suppression of inhibitors upon transplant. Similar findings were obtained for FVIII-specific IgG1 titers (Figure 3C).

Taken together, we conclude that upon the adoptive transfer of re-programmed FoxP3^FVIII^ Treg cells, anti-FVIII inhibitor formation is stably suppressed.

### Combination Therapy With Reprogrammed FoxP3^FVIII^ Cells and Anti-mCD20 Reduce Pre-existing Inhibitors in Hemophilia A Mice

**Figure 4:**
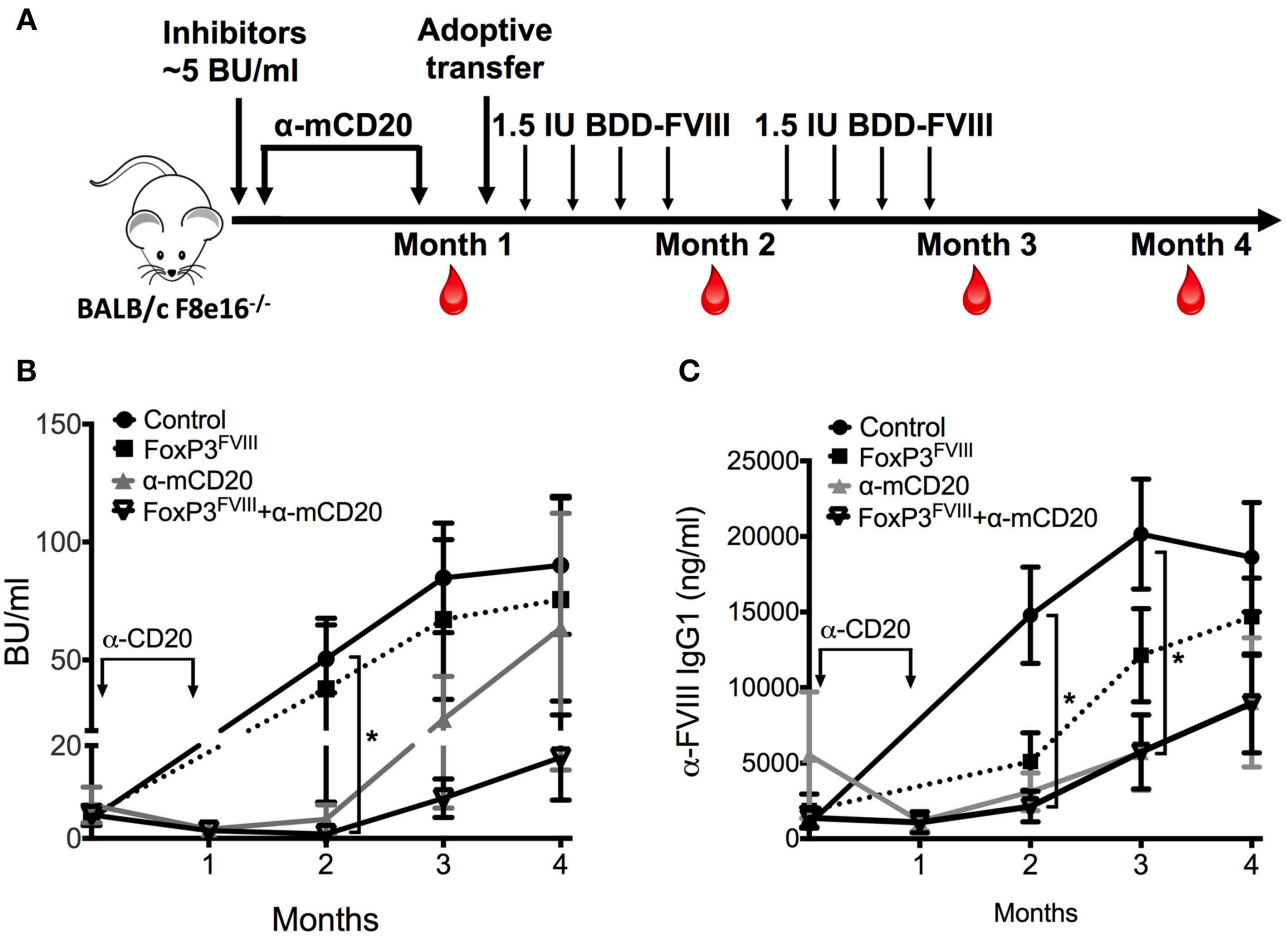
Reduction of pre-existing inhibitors by combination therapy with FoxP3^FVIII^ cells and anti-mCD20. **(A)** Experimental timeline. Inhibitors were established in BALB/c *F8*e16^−/−^ mice by 4 weekly injections of BDD-FVIII. Mice received 2 IV injections of anti-mCD20 spaced 3 weeks apart (α-mCD20 group) following which, inhibitor titers were quantified (Month 1). Another group received 2 × 10^6^ FoxP3^FVIII^ cells 1 week after the last anti-mCD20 injection (FoxP3 ^FVIII^ + α-mCD20 group). Control groups received only FoxP3 ^FVIII^ cells or no treatment (Control). BDD-FVIII administrations were continued for another 2 months following treatment. Blood was collected on Months 2, 3 and 4, respectively. **(B)** Inhibitor titers (BU/ml) measured over time from control and treated mice that received α-mCD20 only, FoxP3^FVIII^ cells only, or a combination of α-mCD20 + FoxP3 ^FVIII^ cells. **(C)** Anti-FVIII IgG1 titers (ng/ml) over time were tested by ELISA. Data are average ±SD. Statistically significant differences are indicated for each time point. ^*^P < 0.05, ^**^P < 0.01, ^***^P < 0.001.

To further evaluate the pre-clinical capability of engineered FoxP3^FVIII^ cells we transferred these cells in combination with B-cell-depleting anti-mCD20 antibodies in Hemophilia A mice which contained previously induced FVIII inhibitors. We previously showed that a short course treatment with anti-mCD20 resulted in a rapid decline of inhibitors, although this effect was transient, and inhibitors rapidly rebounded to their initial titers (37). We therefore tested the ability of FoxP3^FVIII^ cells to tolerize mice with existing inhibitor titers, alone or in combination with anti-mCD20 B cell targeting therapy. As shown in Figure 4A, BALB/c *F8*e16^−/−^ mice (*n* = 5–7) received 4 weekly *i.v*. injections of BDD-FVIII to allow inhibitor development. Mice developed average inhibitor titers of 5 BU/ml, which are considered high titer. This was followed by treatment with either FoxP3^FVIII^ cells, anti-mCD20 (2 *i.v*. injections spaced at a 3-week interval), or a combination treatment with anti-mCD20, followed immediately by FoxP3^FVIII^ adoptive transfer. Mice continued to receive BDD-FVIII injections following treatment.

Mice that received FoxP3^FVIII^ cells developed high titer inhibitors that were comparable to control mice that received only BDD-FVIII injections. Immediately following anti-mCD20 treatment (**Month 1**), both experimental groups (anti-mCD20 and anti-mCD20+FoxP3^FVIII^ group) saw a decline in inhibitor titers (1.67–1.98 BU/ml), which was sustained for another month after resuming BDD-FVIII injections (**Month 2**, anti-mCD20 group: 4.09±3.12 BU/ml, anti-mCD20+FoxP3^FVIII^ group: 0.948±0.42 BU/ml) (Figure 4B). However, tolerance was short-lived, and titers escalated when BDD-FVIII injections were continued at the **Month 3** timepoint (anti-mCD20 group: 24.77±18.24 BU/ml, anti-mCD20+FoxP3^FVIII^ group: 8.65±4.15 BU/ml). As observed, mice that received the treatment combination of anti-mCD20+FoxP3^FVIII^ had lower inhibitor titers as compared to mice that received anti-mCD20 only. This difference was more obvious at the **Month 4** timepoint (anti-mCD20 group: 116.447±104.784 BU/ml, anti-mCD20+FoxP3^FVIII^ group: 22.27 ± 8.015 BU/ml). It therefore appears that whilst anti-mCD20 treatment is effective at short term inhibitor reduction, the re-establishment of inhibitors in response to continued BDD-FVIII injections is more effectively controlled by anti-mCD20+FoxP3^FVIII^ treatment, hinting at a more sustained tolerance mediated by the transplanted engineered Tregs. IgG1 ELISA titers (Figure 4C) were unable to completely recapitulate this difference in functional antibody titers, but this could be attributed to the development of non-neutralizing IgG1 antibodies, which do not interfere with functional activity. Quantification of total B cell, memory B cell, and plasma cell (Supplementary Figure 2) populations from spleen and bone marrow of anti-mCD20 treated mice after the **Month 4** time point confirmed that B cell depletion was transient and the decrease in inhibitor titers at latter time points was not due to generalized immunosuppression. We have earlier shown that immunosuppression mediated by anti-mCD20 is transient and B cell populations in various immune compartments completely recover within 2 months after depletion, while frequencies of T cells remain unaffected by anti-mCD20 treatment [(37); Supplementary Figure 1].

### Conversion of T_eff_ Into Tregs by Reprogrammed FoxP3 Cells as a Putative Mechanism of Lasting Tolerance

**Figure 5:**
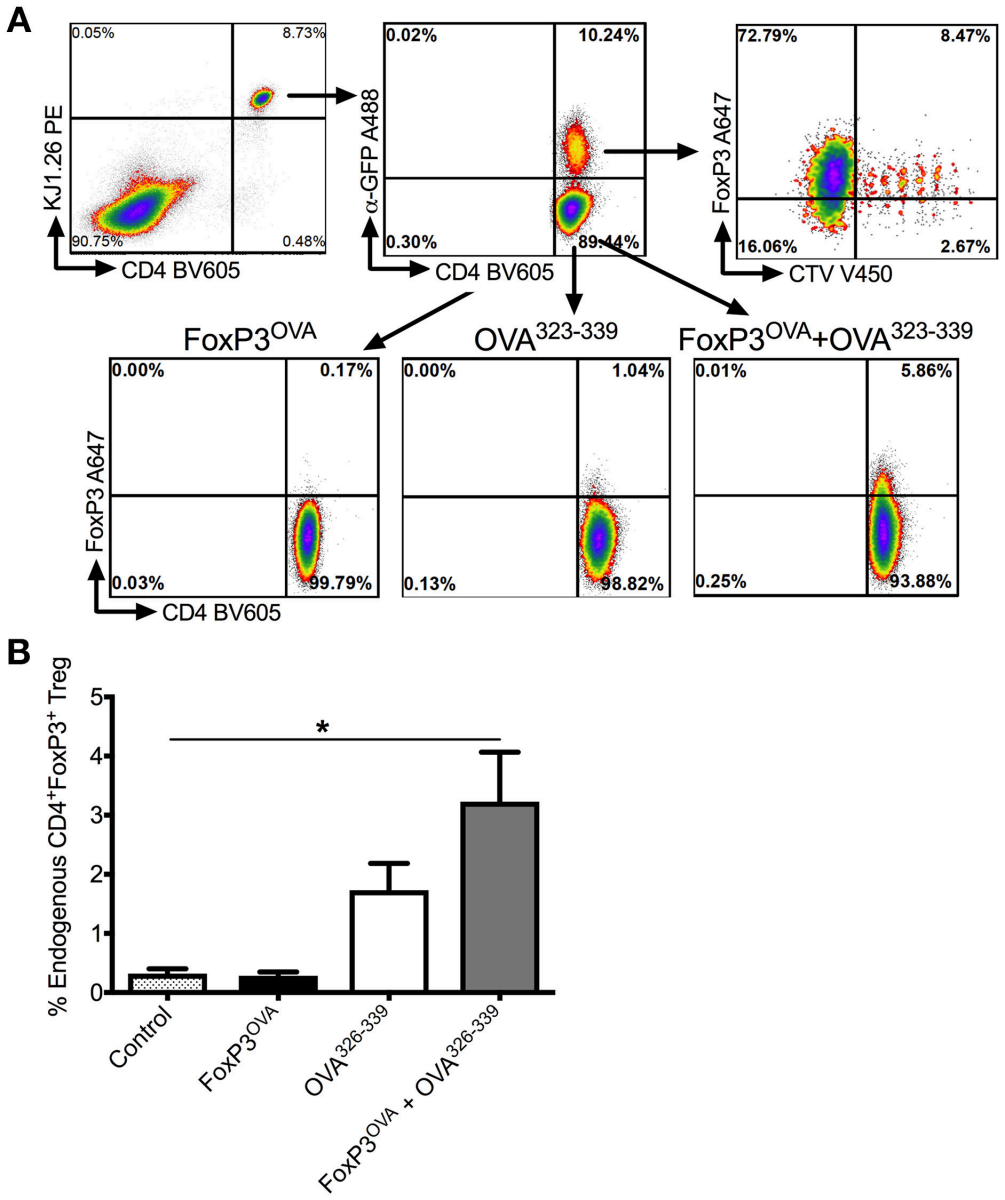
*In vivo* conversion into antigen specific Tregs by transplanted FoxP3 expressing cells. **(A)** Representative dot plots depicting gating scheme. Transplanted FoxP3^OVA^ cells are KJ1.26^+^CD4^+^FoxP3^+^GFP^+^CTV^+^ and thus distinguishable from endogenous *de novo* induced Tregs that are KJ1.26^+^CD4^+^FoxP3^+^GFP^−^CTV^−^. OVA specific FoxP3^+^ Treg induction in DO11.10 Rag2^−/−^ mice is compared between either control group that received no treatment, mice that received FoxP3^OVA^ transplanted cells, administration of OVA^323−339^ peptide only, and mice that received FoxP3^OVA^ cells and OVA^323−339^ peptide, delivered *i.p*. for 2 weeks. **(B)** Bar graphs of induced DO11.10^+^FoxP3^+^ Tregs in treated DO11.10 Rag2^−/−^ mice, indicated as a percentage of total CD4^+^ T cells. Mice received no treatment, FoxP3^OVA^ cells, OVA^323−339^ peptide, or FoxP3^OVA^ cells + OVA^323−339^ peptide. Data are average ±SD of at least 4 animals per group and are a single representative of 2 independent experiments. Statistically significant differences are indicated. ^*^P < 0.05, ^**^P < 0.01, ^***^P < 0.001.

Previous observations by us and others indicated that Tregs that are adoptively transferred into immuno-competent recipients are only transiently detectable for a period of 2–3 weeks (21, 30, 38). Not surprisingly, when we tested a subset of the experimental animals in Figure 3 one-month post-transfer for the presence of donor-derived FoxP3 Tregs, using GFP as an identifier, we were unable to detect any donor cells (data not shown). We therefore looked at the possibility of *in vivo* conversion of host endogenous antigen specific T_eff_ cells into Tregs, which would account for a more long-lasting suppression of FVIII inhibitors. For this, we again used the surrogate CD4^+^ T cell epitope system recognizing OVA^323−339^. We showed that adoptive transfer of CellTrace Violet^+^FoxP3-GFP^+^ DO11.10^+^ CD4^+^ T cells into recipient animals of the same strain was capable of inducing antigen specific Tregs in the recipient mice, which was dependent on the presence of antigen (50 μg OVA^323−339^ peptide injected 3 × /week × 2 weeks) (Figure 5).

As DO11.10 Rag^−/−^ mice lack endogenous FoxP3^+^ CD4^+^ Tregs, were identified with the OVA specific TCR DO11.10 antibody (KJ1.26) and were distinguished from donor DO11.10^+^ FoxP3 transduced Tregs, which were dually positive for GFP and the CellTrace Violet label (Figure 5A). As shown in Figure 5B, transplanting only donor DO11.10^+^ FoxP3 transduced Tregs (FoxP3^OVA^) did not induce conversion of endogenous CD4^+^ T_effs_ into OVA specific Tregs in recipient mice spleens (% DO11.10^+^ Tregs: 0.28 ± 0.06 of total CD4^+^ T cells). OVA^323−339^ administration without FoxP3^OVA^ transfer yielded a small percentage of OVA specific Tregs (% DO11.10^+^ Tregs: 1.73 ± 0.45). These numbers were however, significantly higher in spleens of mice that received FoxP3^OVA^ transfer combined with successive OVA^323−339^ injections (3.228±0.83). Therefore, administration of donor antigen specific FoxP3^+^ cells induced the emergence of endogenous Tregs of the same specificity in recipients, ensuring the persistence of tolerance.

## Discussion

Regulatory T cells are mainly specific for autologous antigens, thus maintaining self-tolerance (39). During autoimmune or inflammatory conditions, the ratio of Tregs to immune effector T cells is perturbed, and an immune response is mounted against self (40). *Ex vivo* expansion of thymic Tregs can increase the population of self-antigen specific Tregs. However, for genetic disorders like severe hemophilia A where significant mutations do not allow expression of the protein of interest, central tolerance often cannot be established resulting in very few antigen specific Tregs.

While the formation of inhibitors during clotting factor replacement therapy has been shown to be dependent on T helper cells, T cell help can in turn be suppressed by Tregs (41), which are instrumental in peripheral tolerance induction. Tregs can also directly interact with and regulate B cells and plasma cells in various niches (42, 43), although this has not yet been demonstrated for hemophilia. Extra thymically induced peripheral or induced Tregs can be FoxP3^+^, LAP^+^ (Th3), or LAG3^+^ (TR1). Of these subsets, FoxP3^+^ Tregs have been the most extensively studied in hemophilia (44, 45). However, this does not discount the role of other Treg subtypes, as LAP^+^ Tregs have been shown to be involved in mediating tolerance to FVIII by oral delivery of antigen (46).

For this study, we reprogrammed antigen enriched T_eff_ cells by ectopic FoxP3 expression, to generate a large pool of engineered cells with Treg function. FoxP3 expression alone has been shown to strongly regulate Treg development and function (47–49) by controlling a transcriptional network of target genes that are involved in promoting the suppressive phenotype among other immunological and non-immunological functions (50–53), as we observed in our studies. FoxP3 transduced cells expressed many Treg associated markers and were strongly suppressive both *in vitro* and *in vivo*. There is valid concern that reprogrammed FoxP3 expressing cells may revert into Th2 cells, or pathogenic Th17 cells *in vivo* (54–56). While this possibility exists, given the plastic nature of T-lineage cells (57, 58), it may depend on attenuation of FoxP3 expression, or may require an inflammatory trigger, which is more common with autoimmune disease. FoxP3 tTregs have also been shown to be remarkably stable *in vivo* and it is possible that this stability extends to peripherally induced Tregs (59). We were able to confirm stable FoxP3 expression in proliferating engineered Tregs *in vivo* after adoptive transfer.

Some pre-clinical studies have been carried out using adoptive therapy with FoxP3 transduced cells, particularly in autoimmune disease and in transplant rejection (33, 60–62). In a few of these studies, antigen specificity has been re-directed by the addition of either a TCR of a single specificity or a chimeric antigen receptor (CAR) molecule upstream of the FoxP3 gene construct (63–65). All of these studies showed efficacy. Using transgenic mice, Jaeckel et al. were able to report that FoxP3 transduction of naïve polyclonal CD4^+^ T cells was not completely effective in suppressing established type I diabetes in mice. Instead, antigen specific FoxP3 transduced cells were highly effective at reversing recent onset diabetes (66). This complements other studies that suggest that antigen specific Treg are superior to their polyclonal counterparts (67–69). Indeed, our data implies that FoxP3 transduction of a population of T_effs_ that are antigen experienced for FVIII and thus enriched for antigen specificity leads to sustained tolerance. The lack of an MHCII tetramer system to identify and isolate FVIII specific Th2 cells in BALB/c mice limits the scope of this study to ascertain the effective minimum dose of antigen specific FoxP3^+^ cells required to suppress an inhibitor response in the hemophilia A experimental model.

For clinical application, the ratios of transduced FVIII specific Tregs and untransduced T_effs_ may vary between patients. However, it is expected that patients with a stronger T_eff_ response would in turn generate more FVIII-specific Tregs. FoxP3 transduction of a mixed population of FVIII specific and non-specific cells may also result in the generation of unwanted Tregs against non-specific antigens. This can be resolved by identifying antigen specific T_effs_ upon short term FVIII specific stimulation *in vitro*. Rapid cell-surface upregulation of activation markers such as CD154 (CD40L) and CD137 (41BB) on antigen-specific triggering (70, 71) allows for detection and isolation of activated cells by cell-sorting before FoxP3 transduction. *Ex vivo* expansion can increase the pool of reprogramed antigen-specific Tregs before infusion.

Among other limitations, the use of the retroviral LTR promoter system for FoxP3 expression has been shown to lead to fluctuations in FoxP3 expression in other studies, depending on the activation status of the transduced cell (33). However, ours is only a proof-of concept study that can be easily adapted to the lentiviral delivery system, as has successfully been shown in PBMC from immune dysregulation, polyendocrinopathy, enteropathy, X-linked (IPEX) syndrome patients, which was able to generate a large pool of FoxP3 expressing Tregs, phenotypically and functionally identical to Tregs from healthy donors (72).

Our *in vivo* studies reveal that a single adoptive transfer with FoxP3^FVIII^ Tregs is able to completely prevent the development of inhibitors, using an antigen dose of 1.5IU of FVIII administered weekly. This was not sufficient to reverse established inhibitors, however, and FoxP3^FVIII^ treatment alone was ineffective to prevent inhibitor escalation in response to continuous FVIII administration. We have observed this in previous studies, where *ex vivo* expanded polyclonal Tregs were unable to completely reverse pre-existing inhibitors to FVIII, although they did halt any further increases in inhibitor titers (30). This is understandable since although it has been shown that Tregs can interact with B cells and plasma cells, it is unclear whether this interaction can lead to a suppressive or cytotoxic outcome. Previously, we have shown that targeting both the B and T cellular compartments by combination therapy with the murine equivalent of rituximab (anti-CD20) and rapamycin (sirolimus) can have a positive outcome in inhibitor reversal (37), which was corroborated in a recent study on a hemophilia B patient with inhibitors (73). On applying this combination therapy to this study, we observed that FoxP3^FVIII^ and anti-CD20 together were superior to either treatment alone and that Treg therapy appeared to prolong the delay in inhibitor re-emergence.

Studies have shown that both murine and human Treg can initiate infectious tolerance, transmitting suppressive capacity from the Treg to the target cell (74, 75). Conversion of CD4^+^CD25^−^FoxP3^−^ T_eff_ cells into Tregs may occur under several conditions, where a number of triggers may induce FoxP3 expression. These may include IL-2 deprivation by Tregs, which reduces the availability of this cytokine for T_eff_ proliferation (76), or the production of suppressive cytokines or receptors by Tregs, such as IL-10 (77), IL-35 (78), TGF-B (79, 80), or CTLA-4 (81). This is also observed in tumors, where tumor antigen specific CD4^+^ T cells in the tumor draining lymph node (TDLN) are activated, but diverted either into anergy or Treg formation, which is enhanced by already present Tregs in the TDLN (82). The complete mechanism is not understood, but strongly depends on Treg: DC interactions in both a contact dependent and independent manner, inducing suboptimal presentation of antigen (83–85). This is usually enhanced by immunosuppressants like rapamycin, which inhibits the mTOR pathway, by interfering with T cell co-stimulation, activation and proliferation (86). We and others have shown that infused Tregs can generate a similar suppressive microenvironment that can promote antigen presentation in a tolerogenic manner, thus inducing the conversion of endogenous T_eff_ cells into antigen specific Tregs (30, 80, 87). In this study, we were able to extend our earlier findings to show that reprogramed FoxP3 expressing cells are capable of inducing *de novo* Tregs of a desired antigen specificity in host animals. We speculate that co-administration of FoxP3^FVIII^ Tregs and FVIII antigen initially generates an immunosuppressive environment due to the high proportion of infused non-specific FoxP3 Tregs. Moreover, the small population of antigen specific Tregs can directly interact with DC to enhance sub-optimal presentation, thus diverting antigen specific T_eff_ cells into Tregs. Tolerance to FVIII protein administration is thus due to a combination of various mechanisms: initial non-specific suppression, interaction of antigen-specific FoxP3 Tregs with DC in the context of antigen presentation, and conversion of Teff cells into endogenous antigen-specific Tregs to confer lasting suppression. Both transplanted FVIII-specific and non-specific Tregs may enhance the latter. Our results imply that a transplant that includes FVIII-specific cells directs a more consistent and durable effect.

Finally, adoptive immunotherapy with Tregs has reported safety and therapeutic efficacy in clinical trials for diseases ranging from autoimmune disorders to transplantation (88). For this treatment to reach its full potential, *ex vivo* expansion of Treg to sufficient numbers of a desired purity needs to be fully optimized. This is particularly challenging in cases where there are perturbations in Treg numbers, or when a desired specificity is required. Engineering conventional T cells to exhibit Treg function and/or antigen specificity therefore has the potential to enhance both Treg numbers and function.

## Ethics Statement

Animals were housed under specific pathogen-free conditions at the University of Florida and treated under Institutional Animal Care and Use Committee-approved protocols.

## Author Contributions

RH, CT, and MB designed experiments, interpreted data, and wrote the manuscript. VK, RS, and MB performed experiments. MB supervised the study.

### Conflict of Interest Statement

The authors declare that the research was conducted in the absence of any commercial or financial relationships that could be construed as a potential conflict of interest.
