# Supplementary material for: Reprogrammed CD4+ T Cells That Express FoxP3+ Control Inhibitory Antibody Formation in Hemophilia A Mice
Source: Front Immunol. 2019 Feb 20;10:274. doi: 10.3389/fimmu.2019.00274 (PMC6391332; doi:10.3389/fimmu.2019.00274)
Supplement: Supplementary file 1 [file Data_Sheet_1.docx]

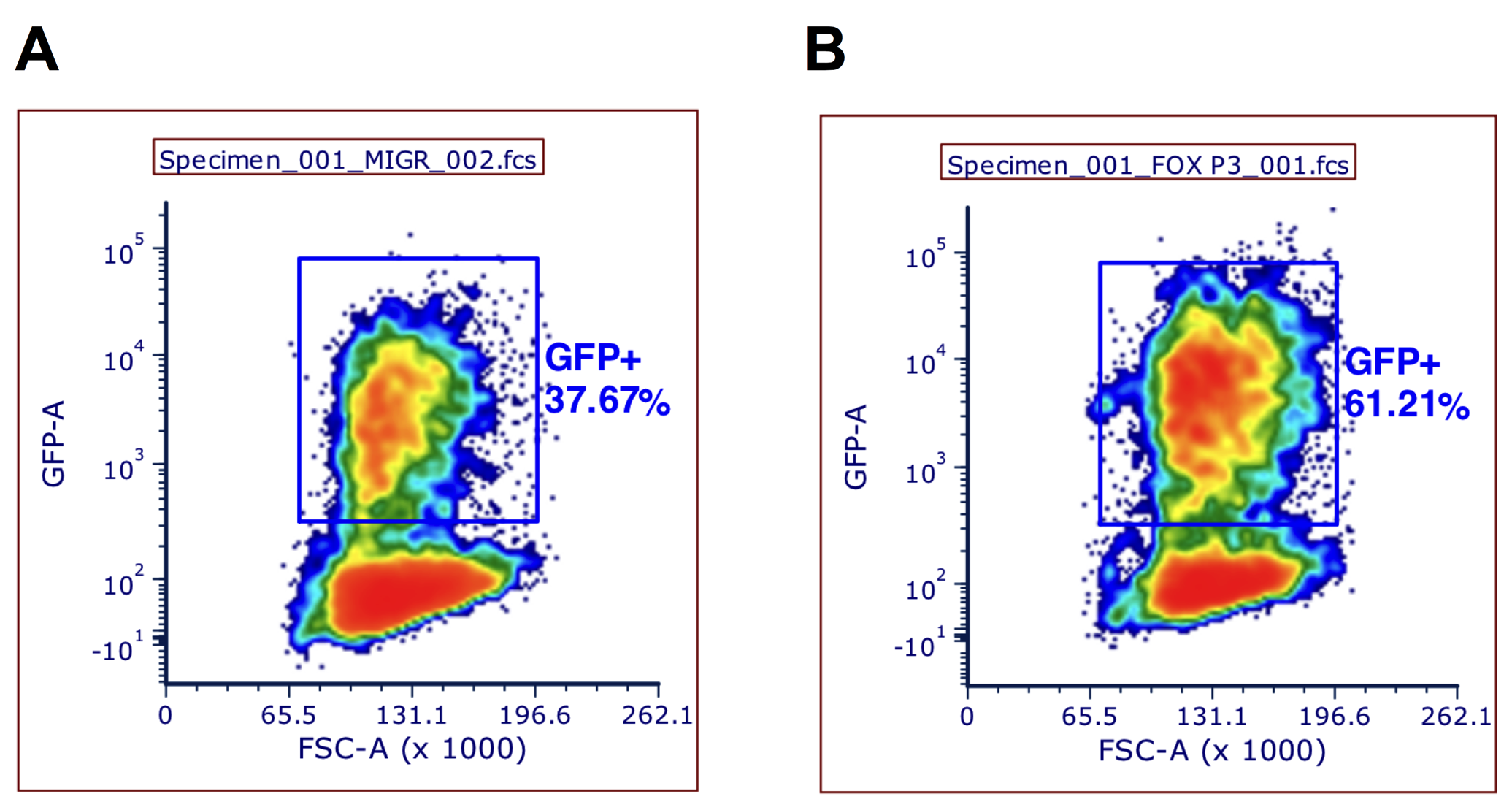


**Supplementary Figure 1.** Transduction efficacy of GFP^FVIII^ or FoxP3^FVIII^ T cells, prior to cell sorting before adoptive transfer.

**
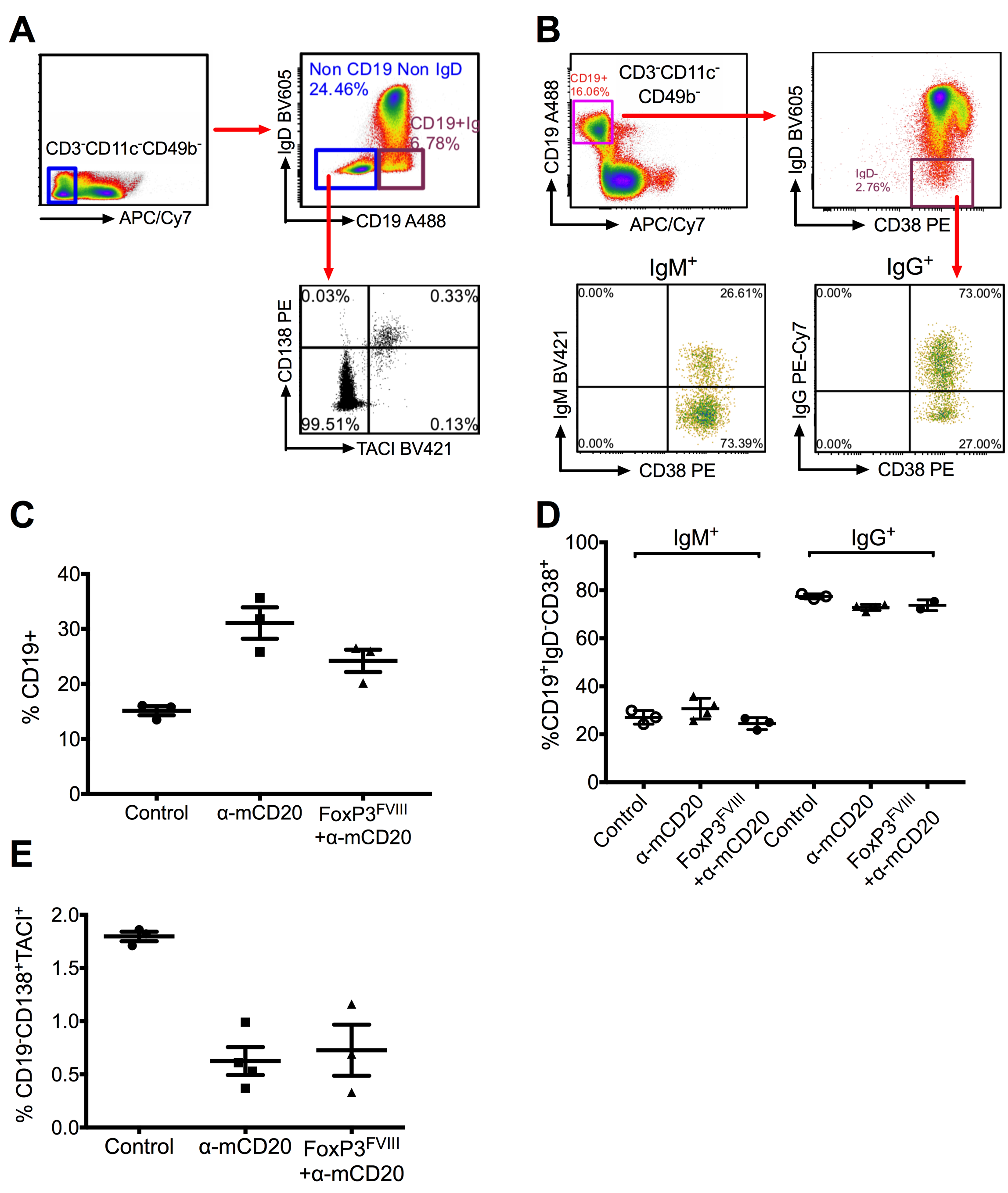
**

**Supplementary Figure 2. Treatment with anti-mCD20 does not affect recovery of B cell compartments.** Spleens and bone marrow of mice treated with anti-mCD20 were harvested after the 4-month time point to confirm that B cell depletion was transient. **A.** Schematic representation of gating for plasma cell populations in the bone marrow and **B.** Memory B cell populations in the spleen. **C.** Total CD19^+^ B cells in the spleens of control, anti-mCD20 treated and FoxP3^FVIII^+anti-mCD20 treated mice. **D.** Percentage of IgM^+^ and IgG^+^ memory B cells in spleens. **E.** Percentage of plasma cells in the bone marrow. Data are average ±SD of at least 4 animals per group. Statistically significant differences are indicated.
